# The disparate effects of bacteriophages on antibiotic-resistant bacteria

**DOI:** 10.1038/s41426-018-0169-z

**Published:** 2018-10-10

**Authors:** Clara Torres-Barceló

**Affiliations:** University of Reunion Island, UMR PVBMT-Saint-Pierre, Reunion, France

## Abstract

Faced with the crisis of multidrug-resistant bacteria, bacteriophages, viruses that infect and replicate within bacteria, have been reported to have both beneficial and detrimental effects with respect to disease management. Bacteriophages (phages) have important ecological and evolutionary impacts on their bacterial hosts and have been associated with therapeutic use to kill bacterial pathogens, but can lead to the transmission of antibiotic resistance. Although the process known as transduction has been reported for many bacterial species by classic and modern genetic approaches, its contribution to the spread of antibiotic resistance in nature remains unclear. In addition, detailed molecular studies have identified phages residing in bacterial genomes, revealing unexpected interactions between phages and their bacterial hosts. Importantly, antibiotics can induce the production of phages and phage-encoded products, disseminating these viruses and virulence-related genes, which have dangerous consequences for disease severity. These unwanted side-effects of antibiotics cast doubt on the suitability of some antimicrobial treatments and may require new strategies to prevent and limit the selection for virulence. Foremost among these treatments is phage therapy, which could be used to treat many bacterial infectious diseases and confront the pressing problem of antibiotic resistance in pathogenic bacteria. This review discusses the interactions between bacteriophages, antibiotics, and bacteria and provides an integrated perspective that aims to inspire the development of successful antibacterial therapies.

## Introduction

Bacteriophages (hereafter referred to as phages) are viruses capable of infecting and killing bacteria. Phages are the natural enemies of bacteria and are present in all ecosystems on Earth, having an enormous impact on microbial communities. Phages are able to insert their own or foreign DNA into bacterial cells, adding genes to already promiscuous and plastic bacterial genomes. By doing so, phages have unexpected consequences for bacterial ecology, including the spread of antibiotic resistance, which is the focus of this review.

**Fig. 1:**
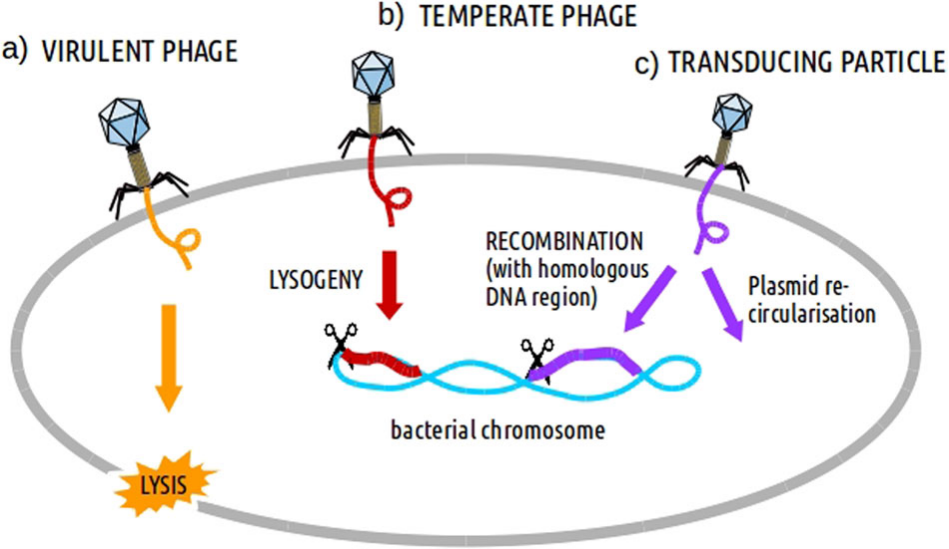
Infection and injection of DNA carried by phages or transducing particles into a bacterial cell. Different processes affecting bacteria can be induced afterwards such as: **a** lysis (by virulent phages), **b** lysogeny (by temperate phages) and **c** DNA recombination/transduction (by transducing particles)

Phages are viruses with a DNA or RNA genome encapsulated in a protein capsid, which is sometimes completed with a tail and more or less complex appendages. Phages attach to specific receptors on the surfaces of bacteria (more than one in many cases) and subsequently inject their genomes into the bacterial cells, after which one of two outcomes may occur. The first is the manipulation of the bacterial metabolic machinery to produce viral proteins and copy the viral genome. Subsequently, the viral particles are assembled and the bacterial cell is lysed, releasing numerous new phages. This is the case for virulent phages (Fig. 1), which only perform lytic cycles, and as a result form a clear halos (plaques) in bacterial lawns. The second possibility is the lysogenic cycle, where phage insert their DNA into the host cell (now called a “prophage”), either as a free plasmid or integrated into the chromosome, similar to human retroviruses, such as HIV. Virulent phages do not enter into this state^1,2^, or if so only transiently as plasmids. Phages able to perform lysogeny are called temperate (Fig. 1). If bacteria reproduce, the daughter cells will also carry the prophage. Phages with the ability to undergo this lysogenic cycle must encode specific enzymes, such as a transcriptional repressor, and if integrated, a so-called integrase. The passive propagation of prophages can be shifted towards the lytic cycle under specific stressful environmental conditions, including the presence of some antibiotics. As a result, the phage genome will be released from the host chromosome, become encapsulated and then the phage particles will be released from the host bacterium, killing it.

Recent advances in genomics have revealed the scope of the impact phages have on bacterial physiology and ecology. Surprisingly, increasing data on these viruses has not always aided our understanding of phages, but raises new questions concerning established paradigms, including the consequences of phage activity with respect to antibiotic-resistant bacteria. Are phages primarily predators (i.e., optimal candidates to control antibiotic-resistant bacteria) or do they serve in bacterial gene exchange processes (helping to expand problematic bacterial genetic capacities) and to what extent? In this review, all these elements are considered together in a general overview of the interactions between phages, antibiotics and bacteria. The integration of multiple perspectives is necessary to design precise therapeutic approaches to better manage bacterial diseases.

### Antibiotic resistance gene transfer between bacteria mediated by phages

It is well established that when bacteria acquire antibiotic resistance, new mutations can spontaneously occur or be achieved via horizontal transfer between cells of the same or different species. Phages are one of the vehicles of this genetic exchange. Antibiotic resistance genes (ARGs) in bacterial chromosomes or plasmids can be mobilized by phages during the infection cycle, a consequence of the inaccurate excision or encapsidation of the phage genome that allows for the incorporation of host genes by mistake. This mechanism, called transduction, was described in 1951 for many bacterial species and soon became a tool for molecular biology used to study bacterial genome architecture, for example see ref. ^3^.

There are two types of transduction mechanisms, generalized and specialized. In generalized transduction, phages can transfer any part of the bacterial chromosome, whereas in specialized transduction only some parts can be transferred. During generalized transduction, the bacterial chromosome is fragmented during phage-induced lysis, and infrequently, a piece is encapsidated forming a “transducing particle” (Fig. 1). These “offspring” phages do not contain phage genes, and only the capsid has a viral origin. Despite this, the transducing particle is capable of injecting the bacterial genes into a recipient cell, which can subsequently be incorporated into the host genome by recombination. This activity implies that external cell receptors must be recognized by the phage and that part of the genome of the receiving cell must be homologous to make recombination possible. It could also be the case that the DNA transduced by the phage was originally a plasmid. Then, it would re-circularize autonomously inside the new cell and become a plasmid again. Generalized transduction can be carried out by both virulent and temperate phages during their lytic cycle. Specialized transduction is typical for temperate phages, which insert their genomes into a particular region of the host chromosome. An inaccurate excision of the prophage can lead to the capture of the flanking genes adjacent to the phage integration point. If capsids carrying the rearranged phage genome with these foreign genes infect other bacteria and integrate into the host chromosome, transduction of the acquired genes will be achieved. The probability that the transferred genes are antibiotic resistance-related is very low.

In the 1950s, the generalized transduction of ARGs in many bacterial species was already being studied^3^. Generalized transduction of ARGs can be demonstrated in the laboratory by exposing antibiotic-resistant (donor) and non-resistant (recipient) bacteria to a common phage that can transfer the ARG of interest. The transduction capacity is then recorded on an antibiotic selective medium as the proportion of the number of bacterial colonies of the recipient strain that can grow on this antibiotic and the total number of phages, expressed as transductants/plaque forming unit (pfu). This method gives a good indication of the transduction capacity of phages, but has some limitations that will be discussed later. Specialized transduction is extremely rare among the transducing cases recorded, partly because of the low probability of ARGs being located in core genome regions, which are the common sites of prophage integration into bacterial genomes. Because the frequency of this type of transduction has been estimated as 10^−9^ transductants/pfu, its impact can be considered to be negligible in most situations. In general, both phenomena occur at extremely low frequencies, as both types of transduction depend on the occurrence of a series of mistakes. However, the possibility of the multitude of phages present in nature spreading ARGs among bacteria is certainly a cause for concern. However current evidence regarding this issue presents ambiguous conclusions, as explained below.

**i) Experimental evidence of antibiotic resistance gene transduction**

The transmission of ARGs by phages has been demonstrated in a great number of bacteria, including *Salmonella*, *Clostridium*, *Streptococcus*, *Staphylococcus*, and *Bacillus* (Table 1)^4–6^. For example, up to 75% of *Streptococcus pyogenes* isolates contain one or more prophages, a large number of which have the potential to perform transduction. Early work from the 1970s estimated that the frequency of generalized transduction is low, from 10^−6^ to 10^−7^ transductants/pfu (Table 1)^4^. In addition to quantifying the rate at which it occurs, it is important to understand when and how transduction events happen in nature. For example, one study showed that a human product released by pharyngeal cells triggers induction (that is, excision from the host chromosome) and generalized transduction of endogenous *S. pyogenes* phages. This in vivo transduction of bacterial genes included one encoding an efflux pump that confers resistance to macrolide antibiotics^7^. This finding is important because *S. pyogenes* is a strict human pathogen, suggesting that phages can potentially spread resistance genes, which needs to be studied in their natural environments.

**Table 1:** Transduction frequencies of different ARGs by different bacterial species

Bacteria	Phage	Antibiotic	Transducing frequency (transductants/pfu)	Interspecies/Intragenus transduction	Reference
*Clostridium difficile*	ϕC2	Erythromycin	10^−6^	Yes	18
*Enterococcus*	EGRM195	Tetracycline Gentamicin	10^−8^–10^−9^ *(tet)* 10^−7^–10^−9^ *(gent)*	Yes	5
*Salmonella enterica* serovar *typhimurium*	ES18	Tetracycline Chloramphenicol	10^−8^ (*tet)* 10^−9^ (*cam)*	No	8
Staphylococcal species	φ80α and φJB	Penicilline Tetracycline	10^−5^−10^−6^	Yes	6
*St*aphylococcus *aureus*	80α	Streptomycin (in SaPI)	10^−1^	Yes	13
*Streptococcus pyogenes*	nd	Tetracycline Chloramphenicol Macrolides Lincomycin Clindamycin	10^−5^−10^−6^	No	4

*nd* non determined

Studies of other bacteria, such as *Salmonella* and Enterococci, are less worrying because of their extremely low generalized transduction frequencies. In separate experiments, the transduction of tetracycline or chloramphenicol resistance was determined to occur at a rate between 10^−8^ and 10^−9^ transductants/pfu (Table 1)^5,8^. The only cause for concern is that for Enterococci, transducing phages were able to transfer antibiotic resistance between different bacterial species^5^. Interspecies transmission also occurs for *Staphylococcus aureus* transduction, including by phages φ80α and φJB, which transfer penicillinase and tetracycline resistance plasmids at moderate frequencies (10^−5^–10^−6^ transductants/pfu) (Table 1)^6,9^. A recent study has revealed that an S. aureus phage was successful at transmitting an active metallo-β-lactamase enzyme, but the activity of the gene after transfer remains unclear^10^. This enzyme confers resistance to a broad range of beta-lactam antibiotics. Additionally, in the notorious methicillin-resistant *S. aureus* (MRSA), methicillin resistance occurs due to a modified penicillin-binding protein located on complex mobile genetic elements, known as SCC*mec*^11^. The strains containing these elements were initially associated with healthcare-related infections and were later identified in community settings, and now they even spread among livestock, posing a major public health threat. Although the transduction of *SSCmec* by phages has been described, the frequency at which it occurs is relatively low, and further studies are needed to assess their real impact on MRSA epidemiology^12^.

There is an important point to be made regarding *S. aureus* specialized transduction. As for other species, bacterial genes spread by phages can be part of particular mobile genetic elements (MGEs). *S. aureus* possesses a family of MGEs associated with virulence and antibiotic resistance determinants that exhibit the highest transduction frequency recorded to date. These transduction frequencies can reach 10^-1^ transductants/pfu, far higher than the highest rate reported for other bacterial species (10^-5^ transductants/pfu; Table 1)^13^. These elements are collectively known as phage-inducible chromosomal islands (PICIs), the prototypical members of which are SaPIs (*S. aureus* pathogenicity islands). PICIs are MGEs that exploit the lytic cycle of specific phages (called helper phages) via replication and dissemination^14–16^. As an example, the transduction of a genetic marker included in a SaPI by phage 80α was 10^7^ higher than that of the same marker integrated at another site in the chromosome^15^. Apart from virulence factors, SaPIs have been shown to carry many ARGs, such as those encoding a penicillin-binding protein, a multi-drug exporter, or those conferring resistance to aminoglycosides, fosfomycin, or fusidic acid^15^. These observations indicate that phages may have a key role in the evolution of MRSA.

Another increasingly problematic bacterial pathogen that can mobilize ARGs is *Clostridium difficile*, in which prophage sequences are ubiquitous. Although previous studies showed that *C. difficile* transposons (another type of MGEs) contain ARGs, whether they could be mobilized by phages was never established^17^. Goh et al. recently showed that erythromycin resistance codified on a transposon is transferred by generalized transduction at an average frequency of 10^-6^ transductants/pfu (Table 1)^18^. However, not all *Clostridium* strains tested were susceptible to the phage or acquired the studied ARG^18^.

**ii) Unclear conclusions regarding the natural occurrence of transduction**

Setting aside the special characteristics of mobile genetic elements associated with phages of *S. aureus*, the frequency of generalized transduction recorded in experimental conditions can be influenced by methodological limitations. Thus, the transfer of ARGs by phages outside the laboratory could be more or less effective than that observed in vitro. There are several reasons why laboratory conditions may underestimate transduction frequencies: (i) potential coinfecting phages may kill transduced bacteria, (ii) phage defense barriers in laboratory strains may prevent the detection of transduction, or (iii) some phages may be unable to be induced (excised from the bacterial genome) in the laboratory. However, the evaluation of transduction in the laboratory using strains that are easy to genetically manipulate typically use prophage-less, plasmid-less, and restriction-deficient recipient strains; thus, the frequency of ARG transduction in nature could be lower than that observed in the lab due to the immunity that some of those factors provide. However, for *S. aureus* plasmid transduction by phages, comparable frequencies have been recorded in phage-sensitive and phage-resistant recipient strains in the staphylococcal population^19^. In summary, to evaluate the role of phages in spreading ARGs, the occurrence of phage transduction is natural environments needs to be determined.

**iii) Molecular methods I: quantifying ARGs**

Advances in molecular biology have allowed phages to be studied without the need for classic microbiology culture-based methods. Rather than estimating the frequency of particles leading to antibiotic-resistant bacteria, it is now feasible to quantify ARG copy numbers in phage populations isolated from various environments. Such frequencies should be ~100-fold higher than observed transductants/pfu values because the final steps of recipient cell infection and the recombination of the ARG gene into the bacterial genome are no longer required. Several recent studies have estimated the presence of selected ARGs in the virome fraction of urban wastewater using quantitative PCR (qPCR)^20–22^.

The results show that *E. coli* ARGs detected by qPCR (e.g., against extended-spectrum β-lactams and fluoroquinolones) in the phage fraction of wastewater matched those identified in the bacterial fraction but were present at lower abundances^20,21^. This method was used to investigate whether the use of sub-clinical concentrations of antibiotics in animal breeding could increase the horizontal transfer of ARGs in soil microbiomes^22^. The ARGs were observed to be abundant in the assayed microbiota, specifically those encoding *E. coli* resistance to streptomycin, sulfamethazine, aminoglycosides, and β-lactams. The quantity of ARGs in the phage fraction did not change depending on the timing or application of manure to agricultural soils, unlike in the bacterial fraction, suggesting that phages regularly harbor ARGs. The Ross and Topp study further isolated phages and experimentally demonstrated the transduction of some antibiotic resistance capacities to other *E. coli* strains^22^. Another team used qPCR to quantify various bacterial genes of an *S. aureus* strain identified in the viral particles of three different phages^23^. This group confirmed the presence of methicillin- and penicillin-resistant viral clones and measured the ratio of encapsidated bacterial genes relative to total phage particles by quantification of a tail fiber gene in phages. Using this method, the authors were able to assess the efficiency of encapsidation, which they determined to a range of 0.0025–0.33%, depending on the phage and the bacterial genes assayed^23^. Phages have recently been shown to even enhance ARG persistence. When measured by qPCR, ARGs remained in phages longer than was observed in bacteria after aggressive inactivating treatments (UV, temperature or pH) in wastewater^24^. The practical implication of these results is that disinfection measures for reducing antibiotic resistance should take phages, as well as bacteria, into account.

As frequently acknowledged by authors, in qPCR studies, the molecular detection of any gene does not demonstrate its functional antibiotic resistance activity or transmission capability, indicating that the transmission of ARGs by phages is likely to be overestimated. This is particularly true when only a gene fragment is quantified, as is common in many studies (e.g., see ref. ^25,26^). The use of various controls to exclude non-encapsidated DNA (which would correspond to free or bacterial DNA) were routinely performed in these studies. However, some degree of bacterial DNA contamination could radically modify the overall outcome, as discussed in the next section. Despite some cautionary reservations regarding frequency estimations, the results of the studies discussed above clearly show that in nature, different ARGs are present in phage particles.

**iv) Molecular methods II: metagenomic analyses**

Another approach to explore the diversity and abundance of ARGs is through the deep sequencing of viral fractions of environmental samples (viromes). A recent study unexpectedly observed an extremely high prevalence of ARGs in viromes when analyzing human and mouse samples. An early study described the higher incidence of ARGs in virome sequences from cystic fibrosis patients than in non-cystic fibrosis viromes^27^. This study highlighted the key role of phages in the evolution of antibiotic resistance, describing the presence of genes related to efflux pumps, fluoroquinolone resistance genes and β-lactamase genes in the viral f7raction^27^.

In a second study, the authors treated mice with ampicillin or ciprofloxacin and sequenced viromes from the collected fecal samples^28^. Relative to control samples from untreated mice, antibiotic exposure caused a 2–3-fold increase in the viral reads annotated as ARGs. Moreover, transductions with the phage mixtures from antibiotic-treated mice resulted in a two-fold increase in antibiotic resistance compared to a naive microbiota^28^. A third study compared the genomes of oral and fecal viral and bacterial communities from humans undergoing antibiotic therapy^29^. While this study reported a significant change in bacterial communities after antibiotic treatment, it did not observe any significant increase in ARG reads among the viral samples from antibiotic-treated subjects.

However, a recent study exposed a methodological problem that inflates ARG counts in metagenomic studies^25^. After re-examining the ARG data from the three studies mentioned above, 90% of the putative ARGs were discarded, revealing that no particular increase in ARG frequency was observed in the viromes of the antibiotic-treated samples studied. Apart from the relaxed thresholds for the in silico detection of ARGs, another factor misleading the conclusions was the presence of substantial bacterial DNA in the viral samples due to weak controls. The authors thus proposed a specific automated algorithm to detect and discard contamination (VirSorter;^26^).

Various studies have not observed ARGs in sequenced prophages, including an examination of 47 *E. faecalis* isolates^30^ and an investigation of 4 phages identified as free viral particles (therefore induced in vivo) and in the chromosomes of *C. difficile* isolated from patient fecal samples^31^. Regardless, the important conclusion, as previously raised by the team that re-analyzed the metagenomic data described above, is that exploratory work on ARGs should continue with the utmost caution, as well as that ARGs are not overrepresented in phage genomes in nature, at least in the samples studied thus far^25^.

**v) Final remarks on phage transduction of ARGs**

A surprising finding was announced in 2006 in *Bacillus anthracis* describing the first antibiotic resistance product encoded directly by a phage. In contrast to bacteria-codified and phage-transferred ARGs, the bacterium acquired phage-encoded resistance to the antibiotic fosfomycin. The authors hypothesize that the gene was of bacterial origin but had been stably maintained in phages as a consequence of the constant antibiotic pressure of fosfomycin-producing *Streptomyces* spp. in the soil^32^. Thus, the selection of antibiotic resistance is a powerful evolutionary force that can lead to such improbable outcomes.

Although transduction is possible, its occurs at a low frequency. For instance, the results of an experimental and theoretical study showed that transduction is probably 1000 times less common than conjugation in bacteria as an ARG transfer mechanism^33^. Undoubtedly, the most important and dangerous case of phages contributing to the spread of ARGs occurs when they are associated with SaPIs. The highly efficient evolutionary strategy of these phage satellites have allowed their expansion to almost all strains of *S. aureus*^15^.

**Fig. 2:**
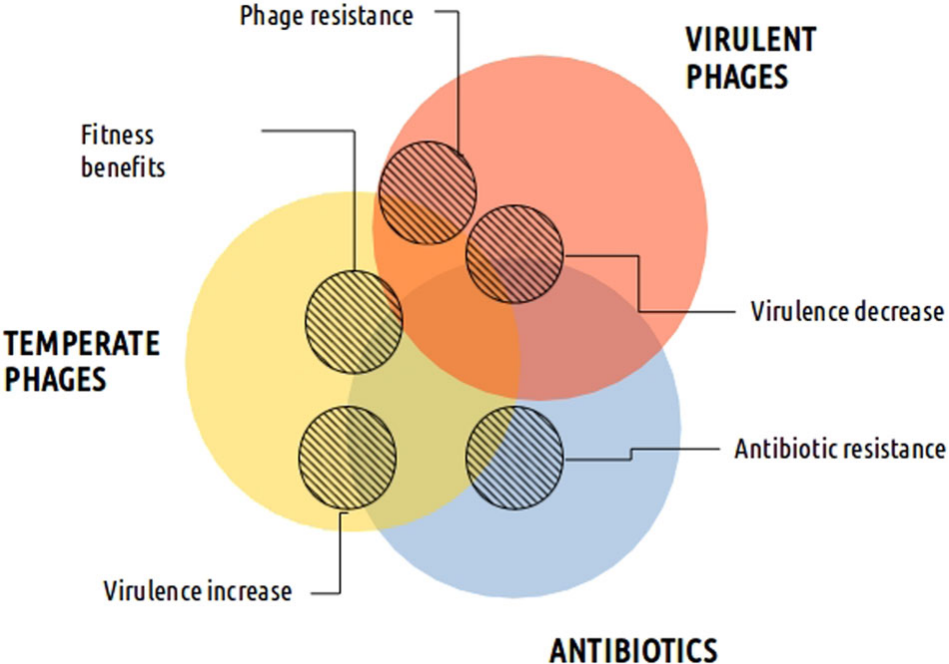
Relative effect of temperate, virulent phages and antibiotics (colored circles) on different bacterial traits as represented by the position of the circles (hatched circles). For example, although antibiotics are primarily responsible for antibiotic resistance, temperate phages also play a role

Sequencing of natural samples is an effective tool to uncover the complexity of natural and clinical processes occurring in pathogenic bacteria. Still, genomic sequencing studies have failed to definitively quantify the genetic contents of phages (Fig. 2). Before raising the alarm, more careful analyses are needed to elucidate the role of phages in the spread of antibiotic resistance. In terms of evolution, the selective advantage for phages to carry these specific genes is contradictory. A potential advantage would be to help their bacterial hosts survive antibiotic exposure. However, because transducing particles harbor defective phage genomes that cannot complete an infection, they are an evolutionary dead-end. Thus, the results of future studies are needed to shed light onto this and other questions.

### Antibiotic induction and mobilization of phages

Prophages are ubiquitous passengers in bacterial chromosomes, as increasingly revealed by genome sequencing projects. They are present in all known bacteria, even in species such as *Helicobacter pylori*, which live in extreme environments where phage infection was initially considered unlikely^34^. Resident phages, ranging from degenerated prophages to functional viruses, can have profound effects on their host physiology and pathogenic potential^35^. Where the previous section discussed how bacterial genes may be mobilized by phages, this section will focus on phage-encoded products. Which factors regulate the expression of these integrated phages? It has been long understood that antibiotics play an important role in the induction of these phages^36,37^. That is, many antibiotics can induce the expression of prophage gene products or lead to the excision and spread of temperate phages. This antibiotic effect may have unexpected outcomes on the virulence of bacterial populations and their resistance to antimicrobial drugs.

The mechanism of phage induction has been well described for model phages, where adverse environmental factors damage DNA in bacteria and activate the bacterial “SOS response”^38^. Bacterial proteins that are activated during the SOS cascade response degrade the temperate phage protein CI, a repressor of the lytic cycle and of the integrated phage genes in general^39^. This response explains why DNA-damaging factors, such as UV light, mitomycin, and antibiotics such as fluoroquinolones and β-lactams, have the unintended consequence of activating and spreading hitchhiker prophages.

**i) Prophages, antibiotics, and virulence toxins**

Stress can induce the expression of prophage genes carrying virulence-related products that would otherwise remain inactive in the genome (Fig. 2). As a result of this process, some antibiotics intended to cure infections can aggravate the health risks associated with pathogenic bacteria. A well-known case of this issue is the Shiga toxin produced by enterohemorrhagic *E. coli* (EHEC) O157:H7 and many other Shiga toxin-producing *E. coli* (STEC) serotypes, which are increasingly resistant to antibiotics^40^. In these bacteria, the production of a toxin encoded in the genome of a temperate phage is dependent on phage induction^36,40^. The phage genome is extensively replicated upon induction, producing the toxin that is released with the lysis of the host bacteria^35^. The intriguing evolutionary implication is that a portion of the bacterial population dies due to prophage induction, diffusing the toxin, while the rest of the population survives, freeing phages to parasitize new hosts. Until now, this has been the only toxin observed to follow this pattern. Shiga toxin-producing *E. coli* O104:H4 cause severe infections that resulted in 51 deaths in Germany in 2011. Sadly, some antibiotics increased the production of the toxin in the treated patients^40,41^. Antibiotics that target processes such as protein synthesis, RNA transcription or protein translation inhibitors have been shown to promote toxin production through other pathways (unrelated to the SOS response), depending on the antibiotic concentration and strain^42^. Additionally, a recent study has shown that the Shiga toxin prophage alters bacterial physiology and increases the tolerance of bacteria to certain antibiotics^43^.

In general, many toxins produced by bacteria are encoded by phages in toxin-mediated diseases, such as diphtheria, cholera, dysentery, botulism, scarlet fever, and some food poisoning diseases^35^. However, not all of these prophages behave in a similar manner. For example, the deadly CTX toxin produced by *Vibrio cholerae* is also encoded by a phage that can be integrated into the chromosome or behave as a plasmid, although the prophage contributes little to the induction of CTX toxin production. In this instance, toxin production is not induced by antibiotics, but is primarily triggered by factors that are mostly encoded in the bacterial chromosome^44^. Moreover, the CTX toxin and phage induction do not result in bacterial cell death because although the phage (CTXϕ, related to the filamentous coliphages) reproduces and is secreted, it does not lyse the host cells^45^. In severe cases of cholera, antibiotic treatment is a good practice that improves patient outcome. With respect to phage-bacterial interactions, it is therefore crucial to adjust treatments so that production of virulence factors is prevented.

As discussed in the previous section, a striking example of bacterial virulence factors and ARGs associated with phages are the *S. aureus* SaPIs, which are implicated in the production of the toxin that causes toxic shock syndrome associated with menstrual tampon use, which caused the death of healthy young women in the early 1980s, among other virulence factors. This SaPI-encoded gene was also one of the first bacterial superantigens to be identified. Other SaPI-associated virulence factors include proteins involved in bacterial phagocyte evasion, the production of enterotoxins and an epidermolytic toxin, and a biofilm-associated protein^15^. Moreover, these pathogenicity islands have a high transfer frequency between different bacterial species and genera^46,47^ and have been identified in other gram-positive bacterial species belonging to other genera, such as *Lactococcus lactis*, *S. pyogenes*, and *E. faecalis*^15^. Although SaPIs are not SOS-induced, their helper prophages are, suggesting that antibiotics can mobilize and potentially spread these traits among bacterial species^48,49^. Alternative treatments to antibiotics are thus advised for bacteria carrying problematic PICIs, coupled with a deep molecular and ecological analysis of the most dangerous pathogenic strains.

**ii) Non-toxin related prophages, virulence, and antibiotic associations**

There are other virulence factors encoded by prophages whose expression is affected by antibiotics (Fig. 2). For instance, in problematic *Streptococcus canis* infections, fluoroquinolone treatments were observed to induce a superantigen codified by a prophage, which likely has a role in the disease^50^. More generally, in the highly virulent *Streptococcus* group A, numerous prophages encode virulence factors, such as a pyrogenic exotoxin (responsible for the rash in scarlet fever and many of the symptoms of streptococcal toxic shock syndrome), an extracellular phospholipase (responsible for the destabilization of membranes and cell lysis) and a streptodornase (DNase that protect the bacteria from being trapped by the DNA webs produced by immune cells)^51^. It was later demonstrated that fluoroquinolone antibiotics could trigger the expression of these products. Interestingly, there is a negative correlation between the presence of inducible prophages and fluoroquinolone-resistance in *Streptococcus*, suggesting that the isolates containing such prophages are lysed during fluoroquinolone treatment before the development of resistance^52^. In a newly published study, the expression of a phage-derived protein was suggested to lead to increased mortality in patients with an invasive *S. pneumoniae* infection, which may be explained by enhanced platelet activation^53^. In addition, a filamentous phage of *Neisseria meningitidis* was recently shown to not be directly associated with increased virulence but rather with greater host-cell colonization, contributing to the invasive disease^54^.

Prophages induced by antibiotics may also trigger problematic side effects by disturbing bacterial communities, such as the human microbiota. For example, children vaccinated with the heptavalent pneumococcal vaccine had increased incidence of *S. aureus*-related acute otitis^55^. It was later discovered that the targeted *S. pneumoniae* normally out-competes *S. aureus* in the nasopharynx. Phages have a role in this clinical issue because the mechanism by which *S. pneumoniae* controlled *S. aureus* was through the production of hydrogen peroxide, a stress factor that induces resident prophages in the staphylococcal host strains, resulting in their lysis^55^. Another example is the growth advantage in terms of gut colonization that integrated prophage-encoded metabolic small RNA regulators provide to problematic EHEC bacteria^56^. However, prophages can also indirectly benefit eukaryotic hosts. *C. difficile* lysogeny by some phages can downregulate major virulence factors of this potential mammalian gut pathogen. In this case, it was suggested that the relationship between phage and host bacteria is mutually beneficial and that downregulation of those specific genes would help the pathogen to overcome the various host defenses in the gut^57^. Therefore, in clinically relevant strains of *C. difficile*, which are generally prophage carriers, antibiotic treatments and phage induction could have antagonizing consequences for virulence^58^. Antibiotics can indirectly increase virulence of part of the *Clostridium* population, while increasing the spread of temperate viruses and decreasing virulence in other sub-populations.

Despite the mobile scenario presented here, prophages frequently suffer massive DNA loss or accumulate mutations that lead to their domestication in the host genome, rendering them uninducible^59^. Some of these defective prophages play a role in antibiotic resistance (Fig. 2) by expressing genes that increase the tolerance and/or resistance levels of the host bacterium. For instance, cryptic prophages were shown to be responsible for protection of an *E. coli* strain against β-lactam and quinolone antibiotics^60^. Inhibition of cell division by the genes encoded by these coliphages appeared to explain the observed increase in bacterial resistance^60^. Furthermore, prophage remnants frequently recombine with temperate or “ex-temperate” phages (temperate phages that have lost genes essential for the lysogenic cycle but retain the ability to exchange sequence fragments with temperate and defective prophages), increasing the risk of propagating virulence factors^61^. Antibiotics have been shown to both induce and repress the expression of defective phage genes, such as in *L. monocytogenes* exposed to different antibiotics^62^, further complicating phage-bacterium-host molecular connections.

**iii) Possible management of prophages and perspectives**

The treatment of bacterial infections in which toxins may be triggered by antibiotics is controversial^40^. As revealed by the recent case involving Shiga toxin genes reaching commensal non-pathogenic *E. coli* strains after exposure to antibiotics^63^, the dispersion of problematic temperate phages among bacterial populations is also of major clinical concern. These unwanted effects of antibiotics should be taken into account, and other therapeutic strategies should be considered immediately.

Prophages encoding virulence factors or ARGs have been suggested to be selected as therapeutic targets to combat bacterial pathogens^64^. This approach is behind a promising strategy to cure AIDS using a recombinase that efficiently removes the integrated HIV virus from infected eukaryotic cells both in vitro and in vivo^65^. Comparing prophages with integrated HIV, targeting prophage integrase has been proposed to potentially be an effective way to reduce the transmission of genes within bacterial populations^64^, although experimental data are lacking. Another possible strategy could involve CRISPR and other bacterial defenses against phages to limit the propagation of ARGs and toxins^66^. Despite growing knowledge of the interactions between phages and bacteria, deep genomic characterization of clinical bacterial isolates is needed to select the most suitable measures to fight bacterial infections, such as the deadly *E. coli* strain O104:H4^67^. Genomic data allows for extremely accurate treatments and the prevention of virulence and antibiotic resistance.

At the same time, antibiotics that do not damage DNA (e.g., protein synthesis, RNA transcription, or protein translation inhibitors) are probably unable to induce the SOS response and are less likely to trigger phage gene expression. Unfortunately, many of the pathogenic strains mentioned are already resistant to most antibiotics. While antibiotics that induce prophages increase the production of Shiga toxin, virulent phages lyse cells and release negligible quantities of the toxin that do not represent a health risk. This result has been demonstrated in vivo in mice, as phages decreased the bacterial load of *E. coli* O154:H4 in the gut with no reported side-effects^68^. Alternative therapeutic approaches are currently available but require scientific and medical support.

### Phages as a solution against antibiotic-resistant bacteria

Virulent phages are perfect bacteria hunters, as they are precise, efficient, self-maintained, and capable of adapting to the strategies of their prey (Figs. 1, 2). Temperate and ex-temperate phages should be obviously discarded for use in developed treatments, as well as phages encoding potential toxins. Protocols for isolating and purifying phages for therapeutic use are simple and readily available^69,70^. There are many reasons why “phage therapy” is increasingly used, and this method could be further exploited. The situation of this type of therapy is opposite to antibiotics, whose efficacy has been radically diminished while new antibiotic types are few or are in the early stages of development^71,72^. In this section, only the specific abilities of phages to deal with antibiotic-resistant bacteria will be discussed.

**i) Coping with antibiotic-resistant bacteria**

Anti-staphylococcal treatments are dwindling due to the selection of methicillin-resistant and vancomycin-resistant strains, which were last resort antibiotics. Fortunately, old and new clinical evidence exists to support the use of phage therapy to fight serious *S. aureus* infections in humans. Numerous virulent phages of *Staphylococcus* described^73^, some of which, such as phage φ812, are able to target hundreds of *S. aureus* strains^74^. The development of anti-*Staphylococcus* phage cocktails has demonstrated that mixtures with only six different phages work efficiently against most common strains (e.g., see ref. ^75^). This efficacy is not the case for other bacteria and makes *Staphylococcus* especially attractive for the broad use of phage therapy. There are accounts of medical use of staphylococcal phages since 1921^76^, and numerous studies performed since 1970 in the Eliava Institute (Republic of Georgia) have reported a significant amelioration of the condition of many patients^77,78^. In other countries, staphylococcal phages have been used to successfully treat infections where antibiotics have failed. In 2016, patients in the US with diabetic foot ulcers infected with MRSA would otherwise have faced amputation if not for phage treatment^79^. The phage used was Sb-1, a staphylococcal phage first isolated in the Eliava Institute that is also effective against *Listeria* and is approved for use in the US. Further studies detailed the use of phages against other antibiotic-resistant bacteria (*E. coli*, *Burkholderia cepacia*, etc.)^66,78–81^. Another case involved the use of phages to successfully treat a patient with an *Acinetobacter baumannii* multiresistant infection^82^. It should be noted that surgical procedures and antibiotics were continued in all these cases. The initial results of extensive double-blind clinical trials in Europe against antibiotic-resistant *P. aeruginosa* will be published soon^83^.

It is also possible to imagine the use of phages as an even more sophisticated and selective therapeutic tool, such as virulent phages that would specifically target antibiotic-resistant bacteria. As previously observed, phages targeting mechanisms associated with antibiotic resistance in bacteria can be engineered^84^, although they are also present in nature. The *E. coli* PRD1 phage uses proteins encoded by plasmids as its entry receptors, so bacteria containing conjugative plasmids are eliminated. Only antibiotic-sensitive bacteria without these plasmids will survive infection with the phage^85^. Another promising example is that of the *P. aeruginosa* OMKO1 phage, which specifically infects bacteria presenting a cell surface protein that is part of the multi-drug efflux system. Phage-resistant bacteria will select for mutated efflux pump mechanisms that will render these structures useless for antibiotic resistance^86^. At least one patient was recently reported to have recovered from a severe infection of an aortic graft thanks to treatment with this phage^87^. The compassionate use of phages in cases such as these could compensate for and reverse antibiotic resistance in bacteria and should be encouraged.

**ii) Targeting pathogenic** ***E. coli*** **while respecting the microbiota**

Phages are so diverse and specific that the appropriate therapeutic phages could be selected to target any problematic bacteria while respecting beneficial ones. The host range of individual phages is usually very narrow, as observed with 20 phages collected from pig stools in *Yersinia* strains^88^. In this study, almost all phages (19) were able to infect more than one *Yersinia* strain, but the majority (13) infected only three different ones of the 94 strains tested, showing a restricted host range. An exception is the vast host range of phage φ812 from *S. aureus* already mentioned, which was observed to infect 95% of 782 *S. aureus* obtained from both laboratory collections and hospitals^74^.

Considering the devastating effect of antibiotics for commensal bacteria, it is obvious that gut microbiota could benefit from the specificity of phages as antimicrobials. Several studies recommend the use of phages as antimicrobials regarding their weak (and most likely indirect) effect on the whole microbial community^89,90^, which has been experimentally confirmed by various studies. First, a study explored the impact of a cocktail of three phages targeting an uropathogenic *E. coli* strain that is increasingly and recurrently provoking antibiotic-resistant infections^91^. The authors demonstrated a reduction of the pathogen in the mouse gut and observed that the impact on microbiota diversity was minimal. Similar results were obtained with the ShigActive™ phage cocktail, which reduced the fecal *Shigella* density in mice after oral administration, with milder effects observed against the resident microbiota^92^.

**iii) Evolutionary capacity: running against evolving bacteria**

The evolutionary capacity of infectious bacteria is a key concern for patients, clinicians and researchers. The mechanisms of bacterial resistance to phages and its consequences for the future of phage therapy have been considered elsewhere in detail^93,94^. It has long been known that an underlying arms-race exists between bacteria and phages. Bacterial populations evolve resistance to phage attack, but phages in turn evolve the ability to infect these resistant bacteria, and so on. This antagonistic evolution has been shown for different bacteria-phage pairs in long-term experiments, such as phage PP01 and *E. coli* O157:H7, phage Phi2 and *P. fluorescens*, and phage RIM8 and the marine cyanobacterium *Synechococcus*^91–93,95–97^.

Evolution has taught us that careful management and prevention of the spread of resistance to any therapy must be a priority. Individualized treatments and the use of phage cocktails could be an effective way to control polymicrobial or phage-resistant bacterial infections, as already shown for *P. aeruginosa* and *Proteus mirabilis* catheter-associated urinary tract infections^98^. The evolutionary capacity of natural phages can be guided in the laboratory before administration to enhance infectivity, expand host range or to promote other desired capabilities, as demonstrated in several interesting studies^99,100^.

Detailed analyses of resistance to phages in bacterial isolates in clinical studies are lacking, but some promising results are available from in vivo experiments. Important pathogenic bacteria have been shown to reduce their virulence following mutations for phage resistance. This is the case with *Flavobacterium columnare* and *F. psychrophilum*, which are emerging pathogens of salmon that cause serious problems in aquaculture. Phage application in vivo reduced the virulence of these two bacteria to become resistant to the phages as a consequence of mutations, such as motility loss and modified cell surface properties^101,102^. Even more indisputable is the example of the cholera-causing bacterium *V. cholerae* sampled from human patient stools from Haiti and Bangladesh^103^. This in vivo study showed that the mutations rendering the bacteria resistant to natural phages were linked to a decrease in virulence and transmissibility of the pathogen. Genetic analyses revealed that the less virulent strains of *V. cholerae* had mutations in *ompU*, which encodes the outer membrane porin and phage receptor. It is expected that treating antibiotic-resistant bacteria with virulent phages may reduce bacterial virulence because additional fitness costs will be imposed on microbes that have already suffered exposure to constant stress. Nonetheless, more research on the evolutionary consequences of phage therapy in different natural scenarios is needed to confirm the encouraging studies described here.

**iv) Phages are more effective when used with antibiotics**

It has been demonstrated that, regardless of the antibiotic resistance state of the bacterium, sub-lethal doses of some antibiotics enhance the infectivity of phages. In particular, they increase the number of viruses produced (plaque size) and decrease bacterial density in a synergistic manner (e.g., see ref. ^104,105^). The mechanism behind the phage-antibiotic synergy (PAS) effect was shown to be associated with the triggering of cell elongation by antibiotics, a trait that benefits phage replication and probably external attachment to the bacterium due to an increase in cell surface^104^. In addition, as recently reviewed, phages and antibiotics may impose different selective pressures and thus have evolutionary trade-offs between resistance mechanisms, or they can improve the control of problematic bacteria through simple demographic combined effects^106^. This beneficial combination has been demonstrated to limit the evolution of antibiotic resistance for many resistant bacteria, such as *P. aeruginosa* and *S. aureus*, in several in vitro studies^107,108^. In vivo experimental evidence also supports the advantages of this particular mixture, such as in protecting birds or mice more efficiently than single treatments^109,110^. In conclusion, phages could prolong the lifespan of so-called exhausted antibiotics, and the application of both antimicrobial agents could also pave the way for the use of phage therapy only, offering the benefits of phages without losing those provided by antibiotics.

## Conclusions and perspectives

This review presents and discusses current knowledge on the consequences of phage activity on antibiotic-resistant bacteria. Numerous recent studies have suggested that phages are important natural reservoirs of bacterial antibiotic resistance capacities^20,24,28^. Others insist that more stringent controls would negate or attenuate this effect^25,111^. The era of high-throughput genomics generates both solid and dubious results. As argued in this review, the horizontal transmission of genes between bacteria mediated by phages is likely to be rare, and better controls are necessary when detecting viral reads matched against known ARGs. An important exception is the high transduction rate of SaPIs observed in staphylococci^16^. This mechanism requires profound consideration and the risk that phages represent needs to be controlled. For the rest of the phage-bacterial world, there is little evidence of the natural occurrence of the phenomenon.

In the realm of bacterial virulence and toxin production, some phages play a clear role. This is true for temperate phages, which codify and spread virulence factors among bacterial populations^35^. Despite this activity, they do not act alone, as antibiotics help them by encouraging their excision and promiscuity between bacteria^42^. This side-effect is one drawback of current antibiotic treatments because it limits the choice of antimicrobials and threatens patient health. However, current genomic knowledge and molecular tools enable an accurate therapeutic approach.

The golden age for medicine to treat bacterial infectious diseases has passed. Virulent phages, natural killers of bacteria, can fill the gap left by antibiotics. The specific advantages of phage therapy have been detailed here. Specificity, unlimited supply, evolutionary capacity and safety are the most obvious benefits of the use of phages as antimicrobials, particularly when compared to antibiotics. Other advantages include the possibility of combining phages with antibiotics to increase their efficacy or to specifically target antibiotic-resistant bacteria^86,106^. As always with phage diversity, positive or negative generalizations are not judicious, but plenty of possibilities remain to be studied. To conclude, careful therapeutic strategies require further multidisciplinary studies to consider the effects of phages on the spread of antibiotic resistance and to demonstrate their potential as antimicrobial agents.
